# Patients who walk out from our Emergency Departments; is alcohol an issue?

**DOI:** 10.1186/1753-6561-6-S4-P56

**Published:** 2012-07-09

**Authors:** B OCallaghan, S Ogorman

**Affiliations:** 1 National University of Ireland, Galway, Ireland

## Introduction

Patients who walk out of the Emergency Department having not waited for treatment (DNW) or against medical advice (LAMA) represent an at risk group in the patient population. Previous literature has attempted to profile these patients under various parameters and to identify the factors which influence their decision to leave. Little to no research has been carried out on the relationship between alcohol and walkout patients. This study aims to profile these patients and examine this specific issue in detail.

## Methods

Patients who Did Not Wait and Left Against Medical Advice over a 1 month period at ED in Letterkenny General Hospital were identified and their charts isolated for review. A proforma sheet was designed and various parameters were recorded from their charts. Patients were followed up by telephone with the aim of obtaining the reasons why they left and to require about any residual medical complaints. Data was recorded using OfficeExcel2007 and analysed using SPSS18.0.

## Results

During the 4 weeks study we found a walkout rate of 2.34%. Single unemployed males in the 18-30 year age group represented the most populous group of walkout patients. 53% of the walkouts had alcohol related presentations such as chronic abuse and/or intoxication. Of these 29% involved violence or an altercation and some 52% had a documented history of psychiatric illness. Some trends were in line with previous literature such as Triage Category and Time of Day. However patient population in the department at the time of admission showed no relationship to the likelihood of walking out. On follow up no patient was noted to have come to subsequent harm.

## Conclusions

This study demonstrates that the reasons why patients walk out may be patient centred rather than based on environmental factors such as overcrowding or staffing issues. This contrasts greatly with previously published literature on this topic. From our results we get a picture of the clinical and social characteristics of the patient who is likely to walkout. In Irish Emergency Departments alcohol is a likely element of the history and presentation of many of our walkouts.

